# Site-Directed Genome Integration via Recombinase-Mediated Cassette Exchange (RMCE) in *Escherichia coli*

**DOI:** 10.1021/acssynbio.5c00031

**Published:** 2025-04-10

**Authors:** Stephan Gutmann, Felix Faschingeder, Christopher Tauer, Karin Koch, Monika Cserjan-Puschmann, Gerald Striedner, Reingard Grabherr

**Affiliations:** † Christian Doppler Laboratory for production of next-level biopharmaceuticals in E. coli, BOKU University, Department of Biotechnology and Food Science, Vienna 1190, Austria; ‡ Biopharma Austria, Process Science, Boehringer Ingelheim Regional Center Vienna GmbH & Co KG, Vienna 1120, Austria

**Keywords:** RMCE, genome integration, synthetic biology, BL21(DE3), homologous recombination, recombinant protein production

## Abstract

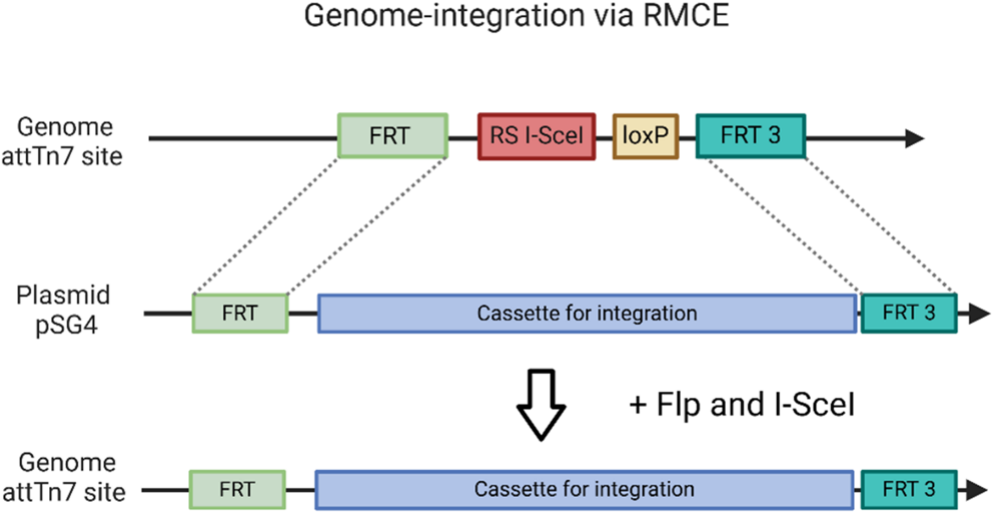

The gold standard for successful genome integration in *Escherichia coli* is the homologous recombination by the bacteriophage-inspired lambda Red system. This method uses the bacteriophage lambda Red recombination proteins to promote homologous recombination between a target DNA sequence and a DNA fragment, which is introduced into the bacterial cell by electroporation. It allows researchers to create specific genetic changes in bacterial genomes, making it a valuable tool for studies in microbiology and biotechnology. However, this system is not without limitations, which are characteristic of its working mechanism and remain to present challenges. The most formidable constraints stem from nucleotide sequences that contain self-homology or homologies to the host genome. These instances lead to uncontrolled homologous recombination events, consequently hindering the desired integration event. Furthermore, handling very large fragments can also be problematic, although, in many instances, this can be overcome by multiple lambda Red integrations in a row. In this study, we illustrate that the limitations associated with the lambda Red system can be overcome through the application of recombinase-mediated cassette exchange (RMCE). This enables the genome integration of larger and more complex DNA fragments and facilitates new research opportunities.

## Introduction

The lambda Red genome integration method, developed by Murphy in 1998,^1^ has revolutionized the field of genetic engineering and bacterial molecular biology. This method allows for precise and efficient modification of bacterial genomes, enabling researchers to easily insert, delete, or replace specific genes or genetic elements. The lambda Red genome integration method relies on three enzymes derived from the bacteriophage lambda, which promote homologous recombination between a target genomic sequence and an appropriately designed DNA cassette.^2,3^ This method has been widely adopted and can be used in various bacterial species, including *Escherichia coli*, to study pathway dynamics and to create mutant strains for functional analysis.^4^ One of the key advantages of the lambda Red genome integration method is its ability to integrate both, single-stranded and double-stranded DNA constructs into a specific genomic target site.^5^ This method eliminates the need for traditional phage integration techniques^6,7^ and allows the precise engineering of genome insertions, duplications, inversions, and point mutations at specific sites within the bacterial genome. The lambda Red genome integration method facilitated the integration of the T7 expression cassette into the bacterial genomes, allowing for the efficient expression of recombinant proteins in bacterial production strains.^8,9^ Despite the numerous advantages, this method also has some limitations and challenges.^10^ One challenge is the requirement of the three proteins, Bet, Exo, and Gam, which must be present and active for a functional recombination process.^11^ While the lambda Red recombinase system is functional in many prokaryotic species, it may not be functional in all bacterial hosts.^4^ Furthermore, this method can be susceptible to off-target recombination events due to the mechanism of the homologous recombination by the proteins Exo, Bet, and Gam.^12^ These off-target recombination events can result in unintended genetic modifications and may affect the stability and functionality of the engineered hosts. A further challenge is the fact that DNA fragments can recombine with any homologous sequence present within the host genome. Therefore, genes or DNA fragments that are already present within the bacterial genome cannot be genome integrated at another, additional locus elsewhere in the genome. This is most probably because of the higher sequence homology at the authentic site, which hinders successful integration at another target site.^13^ Another challenge is the low efficiency of homologous recombination of large DNA fragments. This implies that the rate at which successful recombination events occur is often very low and thus requires elaborate optimization steps. This issue must be addressed by a strong selection system, which allows cells to grow only after a successful recombination event.^14^

An alternative method is the recombinase-mediated cassette exchange (RMCE).^15^ This method enables the site-specific integration of transgenes into *E. coli* by inserting a cassette flanked by self-compatible but mutually incompatible recombination sites at a predefined acceptor locus.^16^ Well-known RMCE systems are the FLP/FRT and the Cre/loxP, which both rely on similar mechanisms.^17,18^ The genes Flp^19,20^ and Cre^17,21^ encode recombination enzymes that recognize and bind to the specific recombination sites FRT and loxP, respectively. In this work, we focus mainly on the Flp/FRT system. For a successful RMCE event to happen, the two FRT recognition sites must be minimally different in their sequences to create a cassette exchange and not the integration of the whole plasmid within the genome.^15^ Multiple mutated versions of these different FRT sites have been generated and experimentally tested for their efficiency by Turan et al.^22^ The mechanism of recombination is explained by the formation of the Holliday junction, cleavage, and ligation by the Flp protein.^20^ This method eliminates the potential issues associated with random homologous integration of transgenes and allows for uniform transgene expression in independent recombinant clones. Moreover, RMCE offers several advantages over conventional transgenic methods using nonhomologous end joining.^16^ These advantages include the predictability, reproducibility, and stability of transgene expression from a single copy integration. Furthermore, RMCE is not limited in fragment size when compared to the lambda Red method. In mammalian cells, fragments up to 120 kbp have been successfully exchanged between a donor DNA and the genome.^23,24^ However, in *E. coli*, the RMCE method is limited to the maximum possible plasmid size, which is normally between 30 to 50 kbp but can reach up to 300 kbp.^25^ In conventional RMCE systems, the donor cassette includes, in addition to the transgene of interest, a selection marker gene and/or reporter protein for cell sorting. Selection markers, such as antibiotic resistance genes, are, however, often avoided, and have to be removed in time-intensive steps. In this work, we employ selection by the homing nuclease I-SceI, which originates in *Saccharomyces cerevisiae* and generates a double-strand break at an 18 bp recognition site.^26^ Homing endonucleases possess long and nonpalindromic recognition sites, spanning 12–40 base pairs, and exhibit coding sequences typically located within introns or inteins.^27^

An I-SceI recognition site (RS I-SceI) does not exist in the genome of *E. coli* and can, therefore, be used as a site-specific selection marker. The RS I-SceI is placed between the FRT and FRT3 sites within the genome, which acts as a landing pad. The presence of the I-SceI protein results in the double-strand breaks of the genomic DNA and the killing of the host, whenever the donor sequence is not exchanged by a successful recombination event.^28,29^ The I-SceI enzyme can be encoded on the same plasmid as Flp, thus the advantages of a single plasmid strategy are achieved. Successful integration could be shown for large fragments, repetitive sequences, and DNA sequences, which were already present inside the genome at another location.

## Material and Methods

### Strains, Media, and Cultivation

For cloning purposes, chemically competent *E. coli* NEB-5α cells were purchased from New England Biolabs (NEB, Ipswitch). For genome integration and I-SceI expression, *E. coli* BL21(DE3)::FRT I-SceI_FRT3 was used. Cells were routinely cultured in Lysogeny Broth (LB) media, recovered in super optimal broth medium supplemented with 20 mM glucose (SOC media), and plated on LB agar. The following antibiotic concentrations were used: Zeocin (Zeo) 25–50 μg/mL, chloramphenicol (CM) 20 μg/mL. Shake flask cultivations were conducted in a semisynthetic medium (SSM) with glycerol as the sole carbon source at 37 °C. In the presence of the pSG4 vector, the temperature was set to 30 °C. Overnight cultures were grown in LB medium supplemented with chloramphenicol (20 μg/mL). For Zeo selection, low-salt LB medium (LBLS) or low-salt LB agar was used. For I-SceI induction, 0.4 M arabinose was added to liquid LB cultures and LB agar plates, respectively.

### PCR

Primers were ordered from Sigma-Aldrich (SA, St. Louis) and Integrated DNA Technologies (IDT, Coralville). For PCR, primers shown in Table S1 were diluted to a concentration of 10 pmol/μL. For amplification of plasmid fragments, the Q5 Polymerase from NEB (New England Biolabs, Ipswich) or the Primestar Polymerase from Takara Bio Inc. (TB, Shiga, Japan) were used. For colony PCR screening, the NEB Onetaq or the TB Primestar polymerase was used.

### Transformation of Electrocompetent and Chemically Competent *E. coli*

Electrocompetent BL21(DE3) cells were prepared as described by Dower et al.^30^ Electroporation was performed in 1 mm gap cuvettes using 50 μL of competent cells. The following settings were chosen: 1800 V, 25 μF, 200 Ω. After electroporation, cells were recovered in SOC-medium.

The cells were propagated by inoculation of 100 mL LB Medium with the *E. coli* strain and incubated at 37 °C. As soon as the culture reached an OD600 of 0.5, the cells were cooled on ice for 15 min. After, the culture was centrifuged at 7000 rpm for 10 min, and the cell pellet was resuspended in 100 mM CaCl2 solution. Afterward, the suspension was centrifuged again at 7000 rpm for 10 min. The cells were finally resuspended in 10 mL of CaCl2 solution containing 15% glycerol. The cell suspension was aliquoted á 50 μL and flash-frozen in liquid nitrogen. The aliquots were stored at −80 °C until further usage.

For transformation, a 50 μL aliquot of competent *E. coli* cells was mixed with up to 25 ng plasmid DNA in a 1.5 mL reaction tube and was incubated on ice for 30 min. The transformation was performed by heat shock at 42 °C for exactly 30 s. Afterward, the tube was immediately put on ice for 1–2 min. 450 μL SOC medium was added per transformation and the cells were incubated in a thermoblock for 1 h at 37 °C and 900 rpm shaking. The cells were plated with different dilutions (undiluted and 1:10 usually worked well) on appropriate antibiotic plates.

### Plasmid Construction of pSG4

The expression system of a pBAD vector containing the Cre recombinase gene under the control of the arabinose promotor^31^ and a Zeo resistance marker was amplified and fused with the origin of replication of the pSIM6^32^ vector. In the next step, the Cre gene was exchanged with the Flp gene, which was amplified from the pCP20 vector with overhang primers and cloned into the target vector by the Golden Gate Assembly (GGA)^33^ method. The FRT_loxP_CAT_loxP_FRT3 sequence was ordered as a gene block from Integrated DNA Technologies (IDT, Coralville) and amplified with phosphorylated primers. The vector was linearized with the StuI restriction enzyme, and the FRT_loxP_CAT_loxP_FRT3 fragment was cloned bluntly at this position. The I-SceI gene was amplified from the pAIO vector created by Egger et al.^28^ and was placed upstream of the Flp gene via GGA cloning.

### Engineering of the BL21(DE3)::FRT_RS I-SceI_FRT3 Strain

A linear DNA fragment was amplified with primers containing 50 bp overhangs homologous to the attTn7 site of the *E. coli* BL21(DE3) genome.^34^ The fragment was purified, and the cassette was integrated into the genome with the pSIM6 plasmid according to Sharan et al.^14^ The antibiotic resistance marker was removed using the Cre-loxP system using Cre recombinase encoded on a pSG1 vector (Supplementary data Figure S6). The final strain was named BL21(DE3)::FRT_RS I-SceI_FRT3, and chemical- and electrocompetent cell stocks were produced and stored at −80 °C.

### Cloning of the Integration-Fragment into the pSG4 Vector

In principle, the DNA insert can be cloned into the pSG4 vector by any given cloning method but the Gibson assembly. We decided to work with the GGA cloning method, which allowed us to use a vector backbone with specific sticky overhangs. This facilitates a fast primer design and cloning of any given fragment. The primers used within this work are shown in the Supplementary Table S1.

## Results & Discussion

### RMCE Toolset for the Integration into BL21(DE3)

To achieve successful RMCE integration, we established a toolset containing three main elements. First, a strain containing the FRT recognition sites at the target locus within the genome. Second, a donor plasmid, containing the DNA cassette for integration, is located between the same FRT sites. In addition, the plasmid carries the genetic information for the recombinase gene Flp under an inducible promotor. Third, a protocol for the usage of the plasmid and strain ensures a highly efficient integration method.

### BL21(DE3) Strain Containing FRT_RS I-SceI_FRT3 Sequence

**Figure 1:**
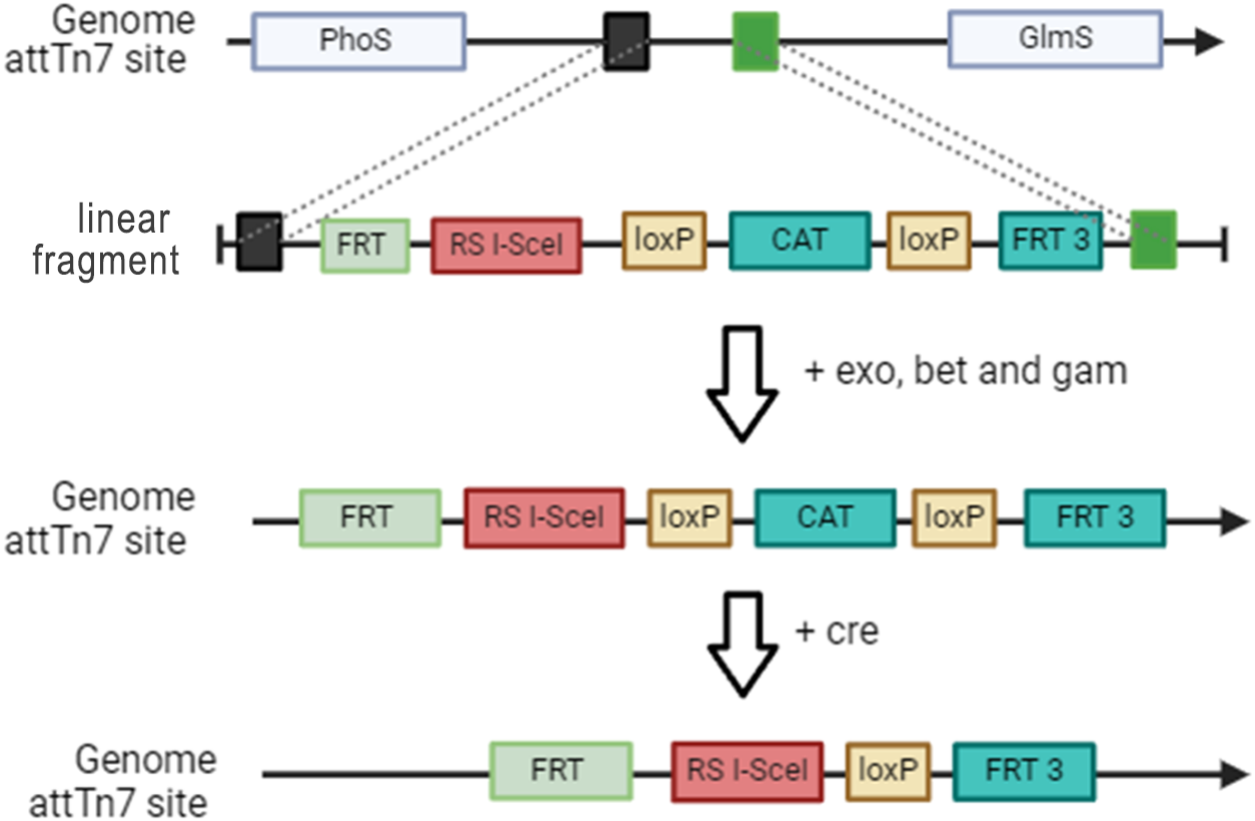
Schematic illustration of the preparation of the attTN7 site in the BL21(DE3)::FRT_RS I-SceI_FRT3 strain by lambda Red homologous recombination.

To enable genome integration using the Flp recombinase, the corresponding FRT recognition sites must be present in the recipient cell’s genome. Therefore, we integrated the two FRT sites at the attTn7 site of the *E. coli* BL21(DE3) genome by the lambda Red system, together with a chloramphenicol resistance gene (CAT), which was subsequently removed using the Cre-loxP recombination system. To facilitate the cassette exchange, the two FRT recognition sites must have the same orientation. Located between the two FRT sequences, is the 18-base-long RS I-SceI, which is recognized and cleaved by the homing endonuclease I-SceI. Subsequently, successful integration was confirmed by PCR and sequencing of the amplified fragment. A schematic illustration of the integrated cassette and the restriction site sequence is shown in Figure 1.

### pSG4 Vector

**Figure 2:**
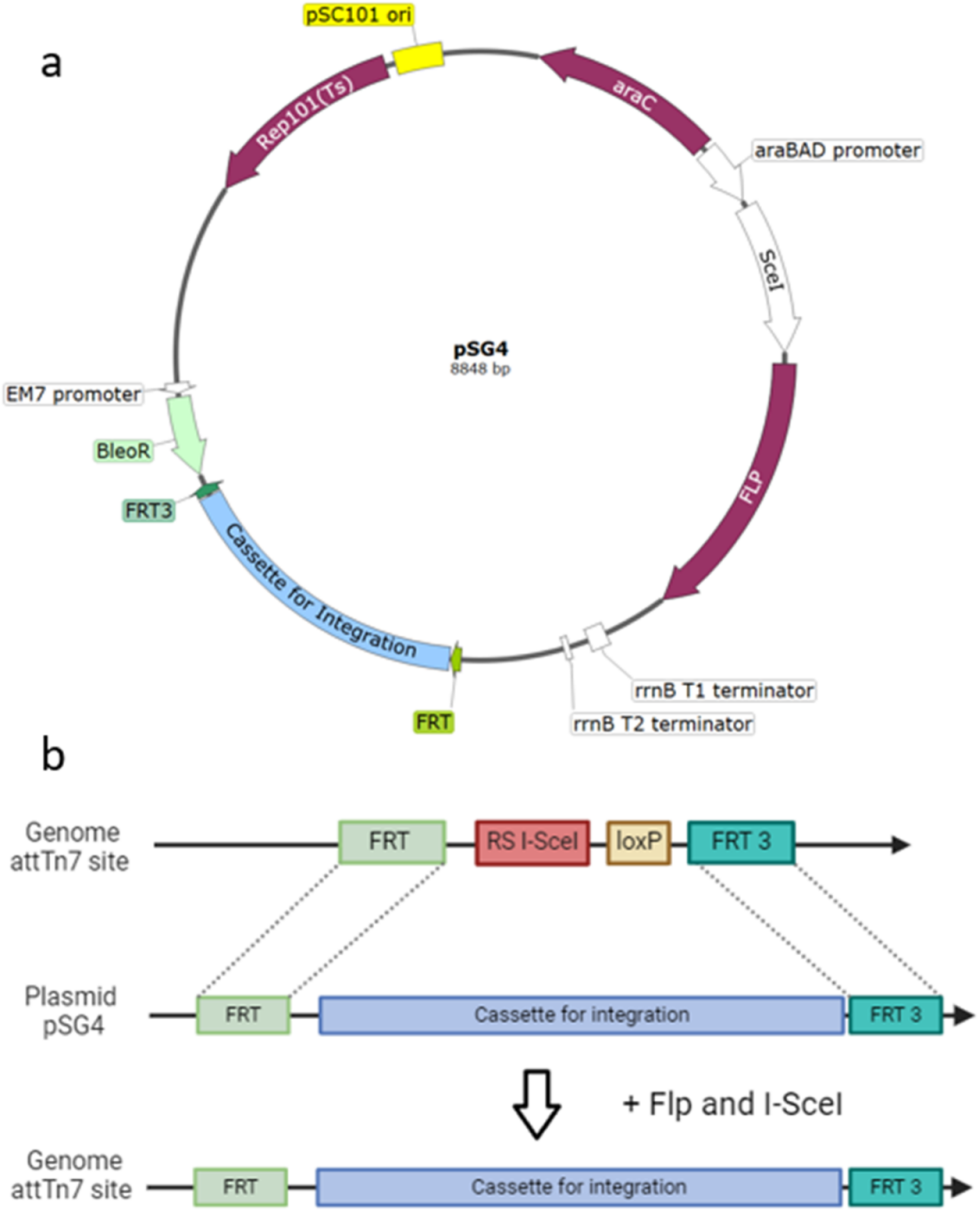
(a) Map of the designed plasmid pSG4, containing the heat-sensitive Rep101 protein, the ORI pSC101,^35^ the antibiotic resistance gene BleoR, and the arabinose inducible polycistronic gene expression system containing the gene Flp and the endonuclease enzyme I-SceI. Behind the two genes, two rrnB terminators are placed to stop transcription. Between the FRT and FRT3 sites, an example cassette for integration is illustrated. (b) Schematic illustration of recombinase-mediated cassette exchange induced by the pSG4 vector; screening primers are located on the genome outside of the FRT sides.

The pSG4 vector was engineered with numerous elements that facilitate the use of RMCE in a highly proficient and effective manner. The plasmid encodes for both, the Flp recombinase and the I-SceI restriction enzyme, both regulated by the arabinose promoter. This setup enables simultaneous integration and selection upon induction with arabinose. The I-SceI gene is positioned upstream of the Flp recombinase gene to ensure minimal leaky expression of the homing endonucleases. The position of genes in a bicistronic expression system showed us a strong influence on leaky transcription (unpublished data). Uncontrolled or leaky expression of I-SceI could cause undesired cleavage of the genome, leading to cellular toxicity whenever no RMCE event was initiated. The vector’s origin of replication, pSC101 combined with Rep101 protein, exhibits heat sensitivity and can be used to cure the host of the plasmid after successful genome integration. The BleoR gene was utilized for selecting the cells capable of growing in the presence of the antibiotic Zeo (Figure 2).

### Genome Integration Procedure

The described protocol can be performed with electrocompetent or chemically competent cells.

**Day 1**: The pSG4 plasmid is transformed into the BL21(DE3)::FRT_RS I-SceI_FRT3 strain and plated on LBLS agar plates containing 50 μg/mL Zeo. The plates are incubated overnight at 30 °C.

**Day 2**: A single colony is picked and inoculated in LBLS medium containing 50 μg/mL Zeo and incubated at 30 °C overnight, but no longer than 16 h.

**Day 3**: In the first step, a cryo sample of the overnight culture is created by a 1:1 dilution with 80% glycerol and is stored at −80 °C in case the integration needs to be repeated.

To start the actual integration, 1 mL of the overnight culture is harvested for 2–4 min at a maximum of 5000*g* and resuspended in 1 mL SSM/glycerol medium added with 0.4 M arabinose (ara) and 50 μg/mL Zeo in a 1.5 mL reaction tube.

100 μL of the cells are diluted 1:10 in the same medium in a 1.5 mL reaction tube. Both undiluted and diluted cells are incubated for 4 h at 30 °C and 800 rpm.

After incubation, the cells are plated at various concentrations on LBLS agar plates, containing 0.1 M ara and 50 μg/mL Zeo. For plating, the diluted cells are further diluted 1:102 and 1:103, and the undiluted cells are diluted 1:103 and 1:104. The plated cells are incubated overnight at 30 °C.

**Day 4**: Single colonies are screened for successful integration by colony PCR. Therefore, the DNA stretch containing the integrated sequence is amplified with TN7 s forward and TN7 s reverse (Table S1). A master plate is created from the picked colonies on LBLS agar with 50 μg/mL Zeo and 0.1 M ara. The plate is incubated overnight at 30 °C.

**Day 5**: One colony positive for successful integration is inoculated into 10 mL LB medium and is incubated overnight at 42 °C. (This is meant to cure the pSG4 plasmid, which has a heat sensitive ORI).

**Day 6**: To obtain single colonies, the overnight culture is diluted 1:101 to 1:107 and plated onto LB agar.

**Day 7**: 25 of the colonies are randomly selected and transferred to LB as well as LBLS + 50 μg/mL Zeo agar plates. The plates are incubated overnight at 30 °C.

**Day 8**: To confirm successful curing of the plasmid in a liquid medium, colonies that grow on LB but not on LBLS + 50 μg/mL Zeo agar plates are inoculated into 10 mL LB medium and 10 mL LBLS + 50 μg/mL Zeo, respectively. The culture is incubated overnight at 30 °C. The cells from the LB culture can now be used to generate cell banks.

To verify correct genome integration, a PCR of the integration site with the TN7 s forward and TN7 s reverse primers can be performed, and the PCR product can be purified and sequenced.

### Proof of Concept for RMCE Method via Integration of a Simple DNA Sequence

For the proof of concept, we selected the reporter gene encoding sfGFP under the control of the T7 promoter system as a model system. This sequence does not contain any challenging elements and can be also efficiently integrated via the lambda Red method (data not shown). The sequence was cloned in between the FRT sites on the pSG4 vector. For simplicity, some sequence elements downstream and upstream of the sfGFP expression cassette were taken directly from the original plasmid and are cocloned, resulting in a total integration cassette length of 2300 base pairs.

**Figure 3:**
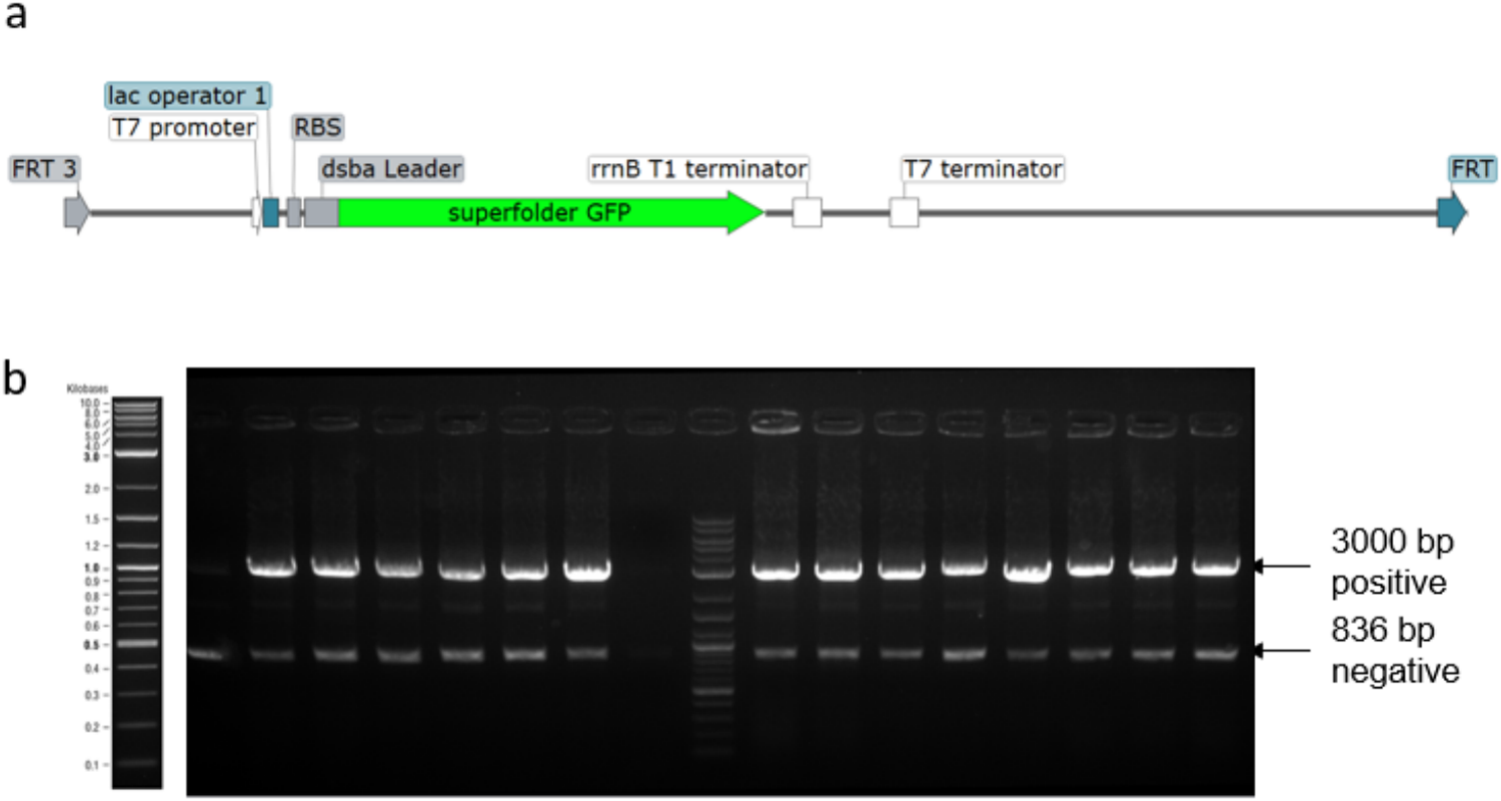
(a) Schematic illustration of the dsfGFP model protein expression construct located between the two FRT sites. (b) Screening of 16 colonies on day 4 of the RMCE genome integration protocol.

Screening of 16 colonies resulted in 14 colonies with a successful integration as shown in Figure 3b representing an integration efficiency of 87.5%. It could be demonstrated that the RMCE method is suitable for genome integration of medium-size genes such as sfGFP into the *E. coli* BL21(DE3)::FRT_RS SceI_FRT3 strain. The integration efficiency was comparable or better when compared to the genome integration methods described by Egger^28^ or Sharan.^14^

The screening in Figure 3b shows that a single-picked colony is a mixed culture of negative cells and positive cells. The subsequent steps (day 4 until day 8 of the protocol) led to pure cultures of positive cells not showing any visible negative bands (Supplementary Figure S1). To verify correct integration, the sequences of the amplified fragments were confirmed by Sanger sequencing.

### Genome Integration of Challenging DNA Fragments

In order to challenge the integration method RMCE, we used multiple DNA constructs, which are not possible to be integrated by lambda Red (data not shown). The sequences selected for this purpose have properties that are responsible for the inhibition of the integration event, namely fragment size, the presence of repetitive sequence segments or sequence homologies to the genome. (Table 1)

**Table 1:** List of Fragments Integrated into the Genome Using the pSG4 Plasmid

construct name	description of integration cassette	size [bp]	challenge of construct	integration efficiency (%)
dsfGFP	model protein sfGFP	2500	proof of concept	93
SMS	large cassette with constitutive promotor (burden to the host cell)	5800	large fragment size	18^a^
Fab1 T7_LC T7_HC	monocistronic FAB expression cassettes	3700	repetitive sequences	100
Fab2^b^ T7_LC_HC+T7_HC	bicistronic Fab with a second monocistronic HC in one expression cassette	3900	repetitive sequences	93
Fab3^b^ T7_LC T7_HC+T7_HC	monocistronic Fab construct with a second monocistronic HC in one expression cassette	3900	repetitive sequences	62
3x (dsfGFP)	large and highly repetitive (three identical expression cassettes in a row)	5300	highly repetitive and large	12^a^
3x (casp_SST)^b^	highly homologue sequence of three times the same expression cassette	4000	highly repetitive	12^a^
3x(casp_PLEC)^b^	highly homologue sequence of three times the same expression cassette	4000	highly repetitive	6^a^
lacI_T7_dsfGFP	model protein sfGFP in combination with the *lacI* repressor gene	3000	homologous with the genome	50

a Indicates that for this construct, a preversion of the final integration protocol was used.

b Indicates that the integration results of this construct are shown in the Supporting Information.

### Genome Integration of a Large DNA Fragment

**Figure 4:**
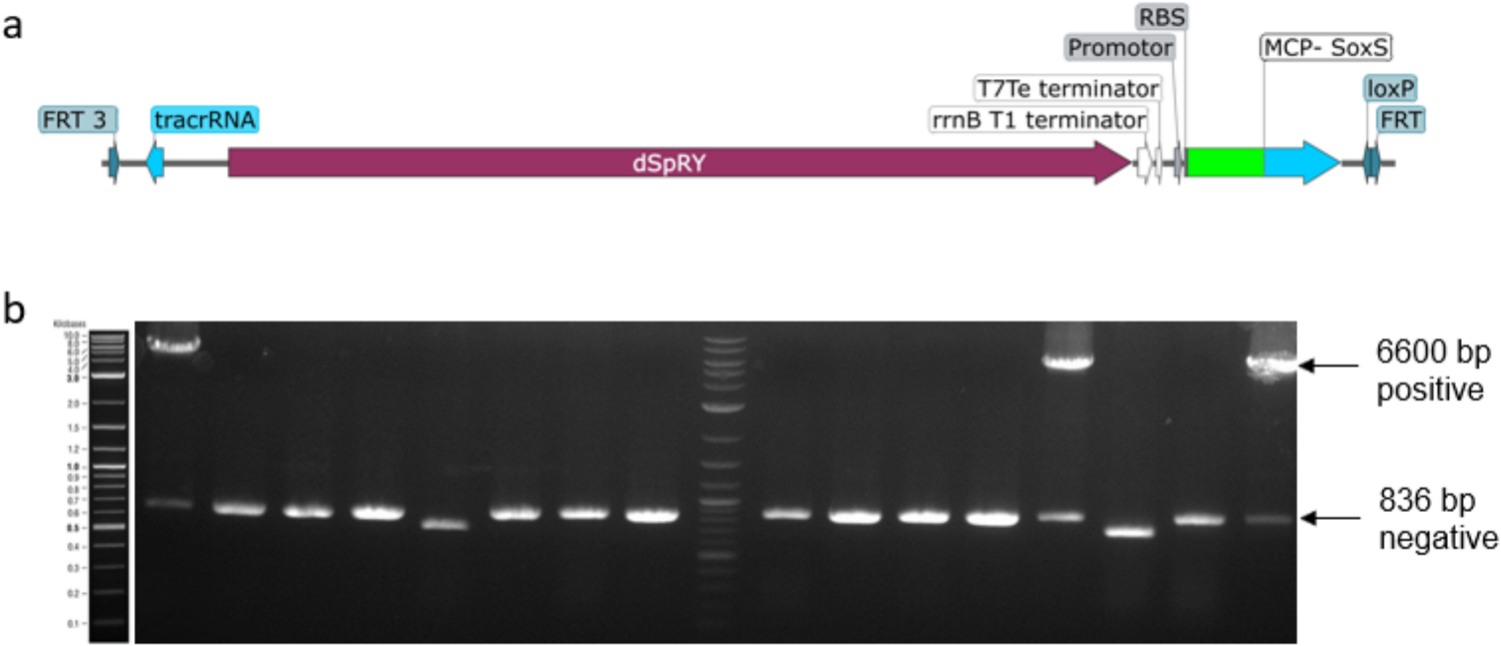
(a) Schematic illustration of the dSpRY-MCP-SoxS expression construct located between the two FRT sites. (b) Screening of 16 colonies on day 4 of the RMCE genome integration protocol resulted in 3 out of 16 positive colonies.

The RMCE method was previously successfully used for genome integration of large segments in animal cells.^36^ In mouse cells, segments up to 120 kbp were exchanged, thereby enabling modifications of larger portions of the genome. However, the fragment size is a key determinant of the integration efficiency, and the integration efficiency decreases with increasing fragment size, but it remains to be seen where the limits lie and to what extent lower yields for large plasmids contribute. However, the RMCE integration is limited in *E. coli* by the size limitation of the donor plasmid and is therefore somewhere between 20 and 50 kbp. In this study, we attempted to insert a DNA fragment consisting of 5800 bps, containing the dSpRY-MCP-SoxS expression construct, a PAMless SpCas9 variant without nuclease activity,^37^ as shown in Figure 4a. Both proteins in this construct are expressed by a constitutive promotor system and result in metabolic burden when placed on a multicopy plasmid (data not shown). Therefore, the integration of a single copy can lower the pressure on the cell and prevent plasmid loss and burden.

The efficiency was at 18%, which is significantly lower compared to the model protein sfGFP. The lower efficiency could be explained by using a not fully optimized protocol in the case of this fragment (the adaptations in the final protocol will be discussed in the next paragraph). However, the integration efficiency is still high enough allowing for low screening efforts requiring only 8 to 16 colonies to identify positive colonies.

### Integration of Fab Fragments with Repetitive Sequences

**Figure 5:**
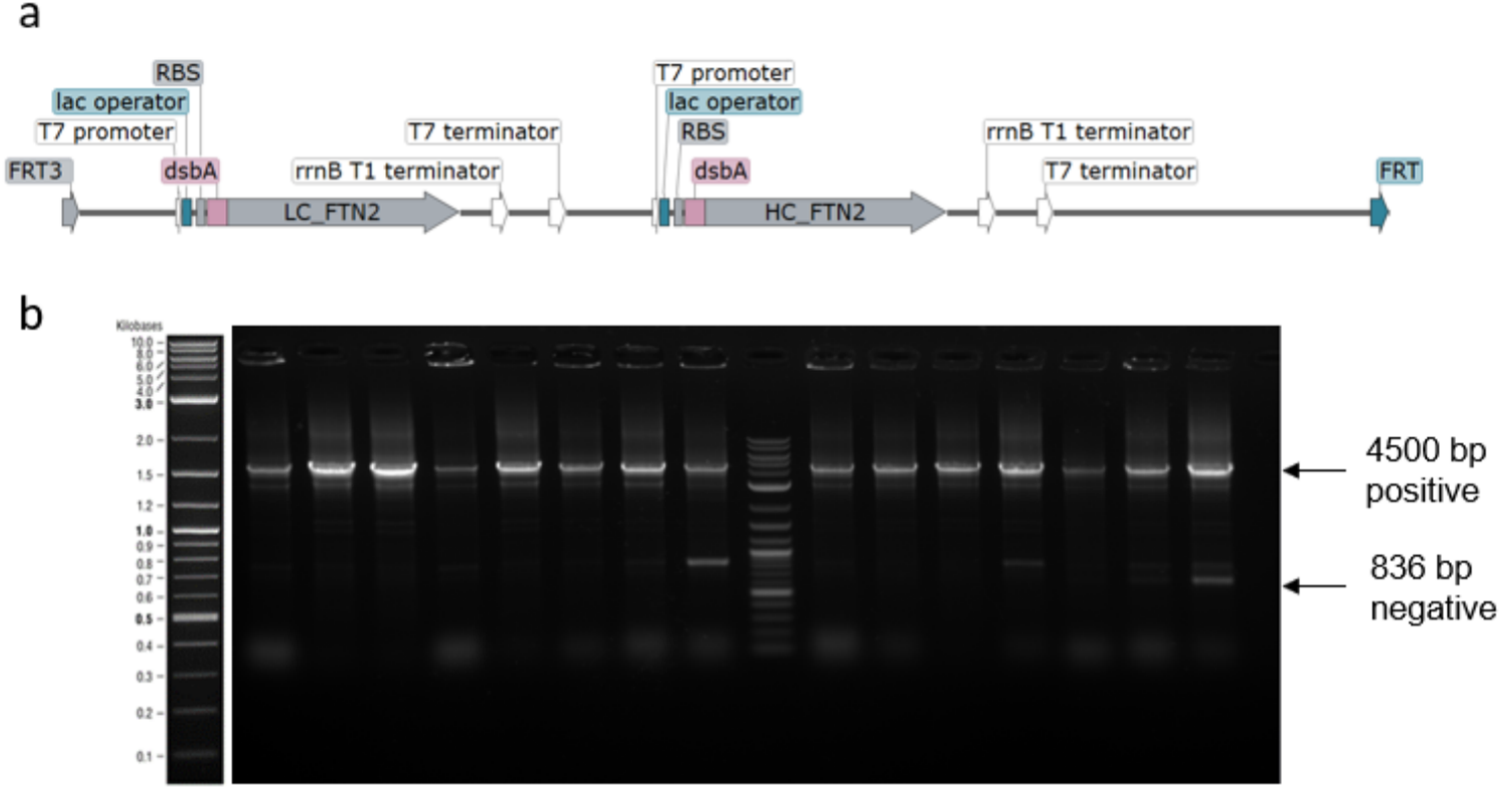
(a) Schematic illustration of the Fab1 light and heavy chain expression construct located between the two FRT sites. (b) Screening of 16 colonies on day 4 of the RMCE genome integration protocol.

The sequence in Figure 5a shows the expression cassette of heavy and light chains for the recombinant production of a Fab in *E. coli*. In most cases, Fabs are produced in bicistronic expression systems, which need only one set of T7 promotor and T7 terminator and result in a single mRNA, which is then translated into two proteins. These systems have shown potential and are commonly used in research and industry.^38^ However, bicistronic mRNA may lead to challenges in the translation, and mostly, mRNA folding impacts the translation efficiency of the second gene.^39,40^

The sequence shown in Figure 5a contains two monocistronic expression systems, which, therefore, need two sets of T7 promotor and T7 terminator. They both contain a lac operator, and the 200 bp sequence upstream of the T7 promotor is kept constant. The cassette to be integrated has repetitive sequences of approximately 400 bases, which was sufficient to prevent the integration of the sequence by a lambda Red system (data not shown).

In Figure 5b, the agarose gel shows the successful integration of the DNA fragment in 16 out of 16 screened colonies. In this experiment, the final version of the protocol, as described in the material and methods section, was used. The final version, which differs only in one step, improved the efficiency and increased the selection of positive cells, which can be understood by the low intensity of the negative band at 836 bp. While using and optimizing the method, we found that oxygen limitation at day 3 during the incubation step for 4 h at 30 °C increases the efficiency. This step was initially performed in a 100 mL Erlenmeyer flask with a volume of 10 mL but was changed to a 1.5 mL reaction tube filled with 1 mL. We assume that oxygen limitation can increase the overall pressure on the cells and therefore reduce the overgrowth of cells escaping the killing effect by I-SceI. The results of the Fab2 and Fab3 versions, which are listed in Table 1, are shown in Supporting Information Figures S4 and S5.

### Integration of Highly Repetitive Sequences

**Figure 6:**
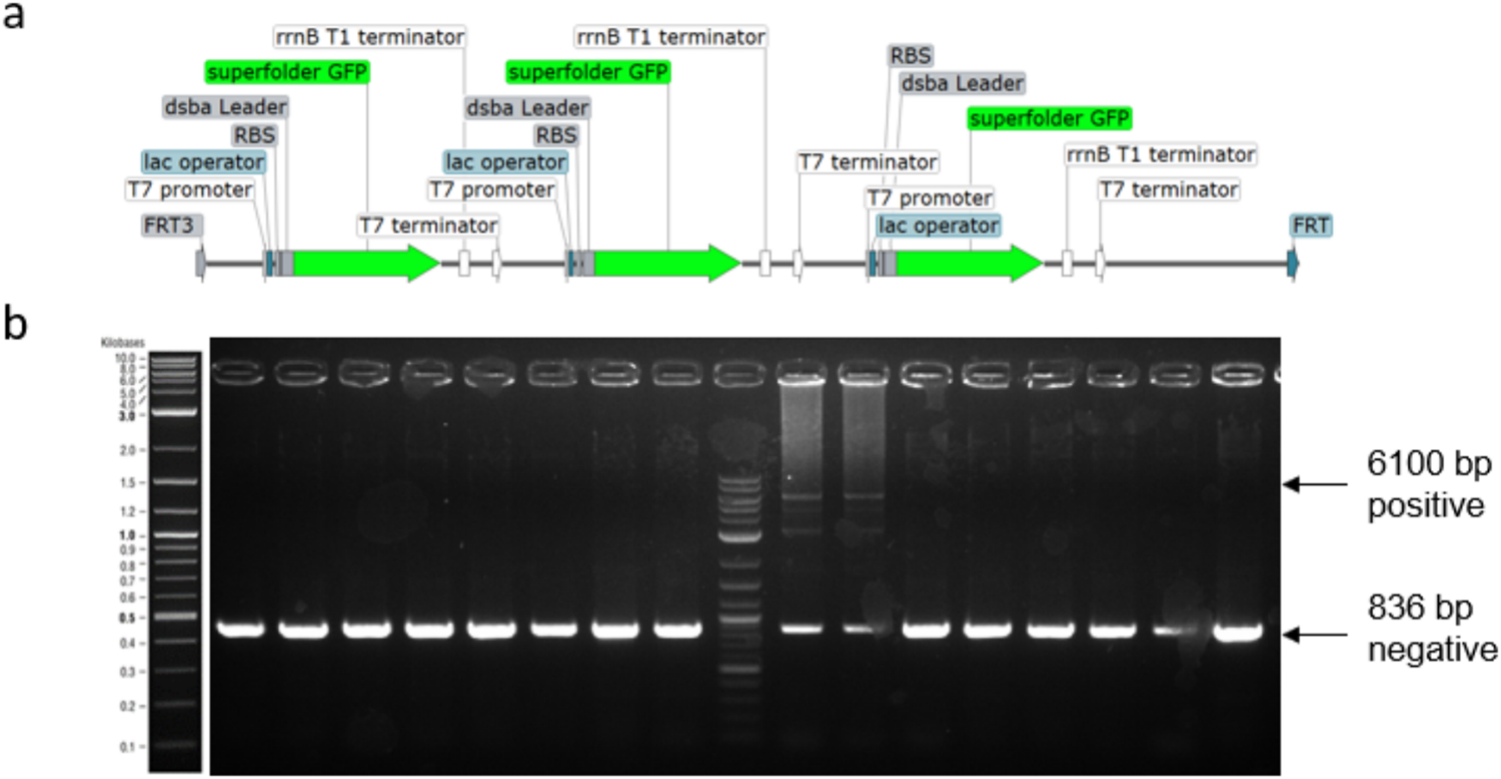
(a) Schematic illustration of triple T7 sfGFP expression construct located between the two FRT sites. (b) Screening of 16 colonies on day 4 of the RMCE genome integration protocol.

The next construct should challenge the RMCE method in terms of a high percentage of repetitive sequences. We designed and cloned a DNA fragment consisting of three identical expression cassettes (Figure 6a). Each of the three sfGFP cistrons contains one T7 promoter, one lac operator, and one terminator. Also, the 200 bp upstream sequence of the T7 promotor, which originated from the pET30a vector, was kept constant for each expression cassette. In addition, the CAT selection gene was included downstream of the third sfGFP gene. This results in a total sequence length of 5300 base pairs, of which around 90% were repetitive. To generate this highly repetitive fragment within the pSG4 vector, we used the GGA cloning method.

The integration efficiency of the construct was significantly lower when compared to the single sfGFP construct (shown in Figure 3a). Two out of 16 clones tested positive as shown on the agarose gel in Figure 6b. The positive bands were less intense when compared to the negative bands. Additionally, multiple bands between the 6100 and 800 bp are visible. We hypothesized that the high number of repetitive sequences was interfering with the PCR by forming loop structures. The construct was mainly designed to challenge the integration method but was also tested in microtiter cultivation and showed only low increases in fluorescence when compared to the single sfGFP construct shown in Figure 3a (cultivation data not shown). We assume that the cells are already limited in production capacity when induced with a single copy of sfGFP or that higher expression levels led to increased inclusion body formation with much lower fluorescence signal. This assumption is supported by the fluorescence level of similar high copy plasmid expression systems. Next to the sfGFP gene also, two peptide sequences have been tested three times in a row (listed in Table 1). In both cases, the peptides as well as the promotor and terminator region are tripled. The results are shown in Supplementary Figures S2 and S3.

### Integration of a Fragment with Homologies to the Genome of BL21(DE3)

**Figure 7:**
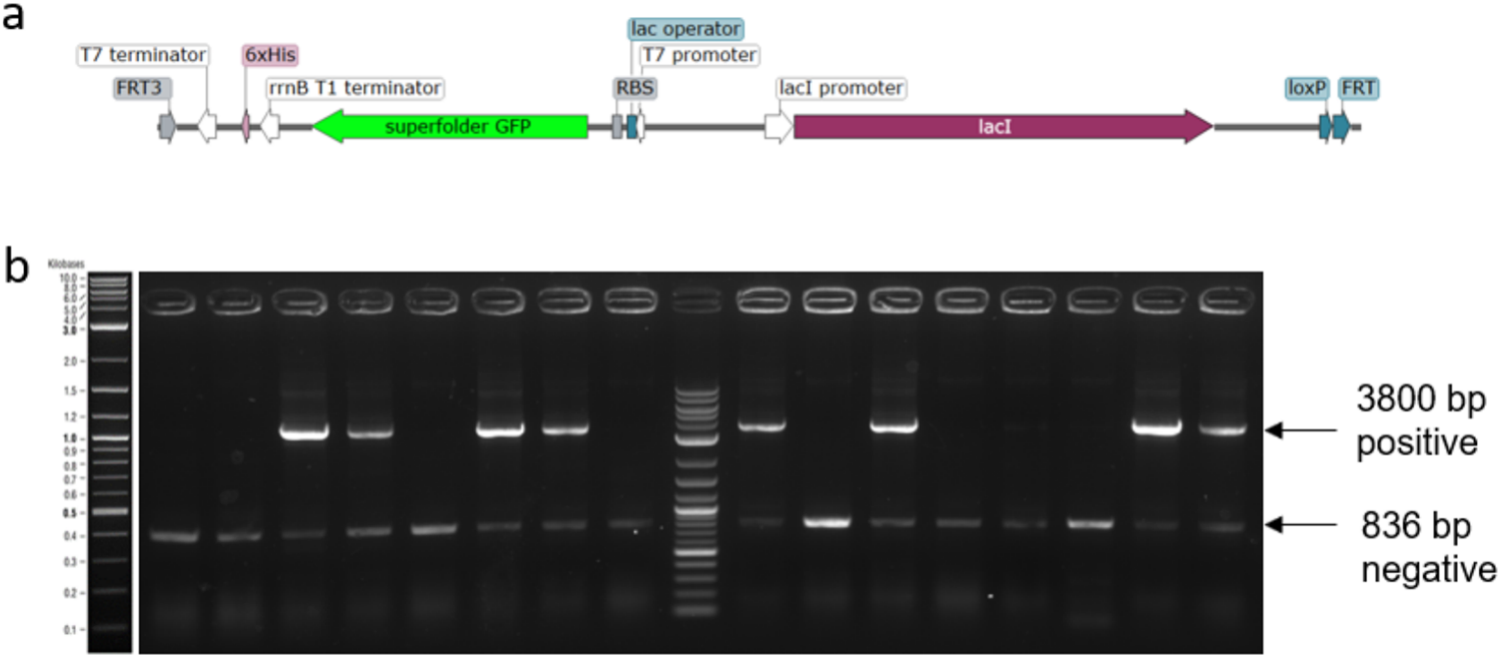
(a) Schematic illustration of the T7 sfGFP expression construct and the lacI repressor gene located between the two FRT sites. (b) The screening of 16 colonies on day 4 of the RMCE genome integration protocol.

With the last fragment, we demonstrated that this method facilitates the integration of sequences homologous to the genome of the host at another distinct location. We engineered a construct containing the model protein sfGFP in combination with the lacI gene (Figure 7a), which is also present within the genome of BL21(DE3). This makes the lacI gene a suitable candidate to challenge the RMCE method in this respect. The lacI gene is under the control of the natural constitutive LacI promotor. When introduced to the genome, we assume that the LacI protein level is increased and therefore lowers the leaky expression of the T7 polymerase in the BL21(DE3) strain. The lacI gene used in this construct was amplified from the pET30a plasmid, which also contains the lac operator 3 upstream of the lacI gene.^41^ Therefore, the LacI expression is self-regulated and increases the amount of LacI only to a limited extent.

In Figure 7b, the agarose gel shows the successful integration of the fragment in 8 out of 16 screened colonies. The integration was performed by the final protocol described in this work. The designed pSG4 plasmid and the optimized protocol facilitate the integration of host homologous DNA fragments in an easy and time-efficient manner.

## Conclusions

Site-directed integration of target genes into *E. coli* expression host cells is one of the key tools for efficient cell design. Usually, linear DNA fragments flanked by homologous sequences for site-specific integration are provided and cointegrated antibiotic resistance genes serve as selection markers.^14^ The combination of the lambda Red system and the CRISPR/Cas9 selection system has previously enabled the integration of larger and more complex constructs.^42^ However, both systems may induce sequence-unspecific off-target effects, resulting in changes within the genome at other positions, often difficult to identify. Furthermore, the ongoing patent disputes surrounding the CRISPR/Cas system pose a challenge to its industrial applicability.

Integration of DNA fragments becomes more challenging when they increase in size, contain repetitive sequences, or are homologous to sequences elsewhere in the *E. coli* genome.^13^ We demonstrate that genome integration in *E. coli* via the RMCE method is highly suitable and can overcome the intrinsic limitations of the lambda Red system. When working with linear DNA fragments, the integration efficiency depends on the transformation efficiency of the linear fragment and can differ between each experiment and fragment type. Due to the combination of the selection system based on I-SceI with the recombination step, we could achieve very high efficiency and establish a simple protocol. An additional benefit is that the fragment does not require a separate introduction as either single-stranded DNA (ssDNA) or double-stranded DNA (dsDNA); instead, it resides on the same plasmid intended for integration and transforms in tandem. This combination of favorable characteristics within this new method renders it an exceptionally efficient and time-conserving tool in the field of genome engineering.

All fragments were integrated at the same position, the attTn7 site, of a previously generated *E. coli* strain. A medium-size fragment of 2300 bp (Figure 4) was integrated successfully and was shown to be comparable to the lambda Red method in terms of efficiency and proved the functionality of the RMCE method in *E. coli*. Integration of large sequences or repetitive sequences is quite challenging, presumably due to secondary structures within the DNA and other factors. Yet sometimes, the same promoter is desired for several genes resulting in homologue sequences within the fragment to be inserted (Figure 5). In Figure 6, we were able to demonstrate that sequences, which are up to 90% repetitive, can be genome integrated by the described RMCE method. This means RMCE is a useful application in synthetic biology and can enable the fast integration of highly complex sequences within the genome of *E. coli*. However, it is well-studied that naturally occurring repetitive sequences are prone to rearrangement and deletion.^43^ Therefore, the stability of the mentioned sequences could be conducted in further research by cultivation in standard fed-batch experiments.

Also, other methods such as Flp/FRT-^44^ or Cre-Lox-based^45^ integration methods have been developed for *E. coli*, and methods relying on selection on I-Sce-I have been adapted in *E. coli*.^28^ However, the combination of both was not demonstrated, and our method enables the integration of large fragments with repetitive sequences as well as for fragments that contain homologies to genomic sequences elsewhere on the chromosome.

We present an optimized protocol for genome integration, suitable for complex and difficult to insert DNA sequences based on the use of RMCE. The integration plasmid, pSG4, is a highly rational designed and easy-to-use vector and will be available for research at the BOKU materials Web site (https://materials.boku.ac.at). We showed successful integration of a series of challenging DNA constructs with a very low screening outlay of 16 colonies. The adaptation, optimization, and implementation of the RMCE technique contribute to the variety of genetic engineering tools. Yet, the integration of even larger DNA fragments (>6000 bp) must be tested as well as other bacterial strains to expand the versatility of this method to a wider field of applications.

## Data Availability

The pSG4 plasmid will be available at BOKU materials (https://materials.boku.ac.at).

## Supporting Information Available

List of primers used in this study; schematic illustrations of additional sequences used for integration; additional agarose gel images; vectors/plasmids constructed and used in this study; DNA sequence of sfGFP used in this study; and schematic illustration of the pSG1 vector (PDF)
