# Supplementary material for: Site-Directed Genome Integration via Recombinase-Mediated Cassette Exchange (RMCE) in Escherichia coli
Source: ACS Synth Biol. 2025 Apr 10;14(5):1667–76. doi: 10.1021/acssynbio.5c00031 (PMC12090343; doi:10.1021/acssynbio.5c00031)
Supplement: Supplementary file 1 — sb5c00031_si_001.pdf [file sb5c00031_si_001.pdf]

Supporting information

Site-directed genome integration via recombinase-mediated cassette exchange (RMCE) in *Escherichia coli*

Stephan Gutmann[1], Felix Faschingeder[1], Christopher Tauer[1], Karin Koch[2], Monika Cserjan-Puschmann[1], Gerald Striedner[1] and Reingard Grabherr[1]*

[1] Christian Doppler Laboratory for Production of Next-Level Biopharmaceuticals in *E. coli*, BOKU University, Department of Biotechnology and Food Science, 1190 Vienna, Austria.

[2] Biopharma Austria, Process Science, Boehringer Ingelheim Regional Center Vienna GmbH & Co KG, 1120 Vienna, Austria.

[*] monika.cserjan@boku.ac.at

Supplementary Table S1: List of Primers

Name	Sequence
SceI_RS_insert_for	ttgGGGCTAGCAGGAGGATA
SceI_RS_insert_Rev	AAAAAAACCTCCTATTTTCGAGACTCTGGTGAATTCCTCCTACACGAATTC
I-SceI for	CAGGGTAATCGCCTCGAGATAACTTCGTATAG
I-SceI rev	TTATCCCTACGCGCAGGTGGAAGTTCCTAT
pSG1_his_Rev	P-GTGGTGGTGATGATGATGTGCCA
pSG1_SacI_sense	gcagagctcCCATTAACGCGTAAATGATTGCTATAATTAGTTG
FLP sense	P- ATGCCACAATTTGGTATATTATGTAAAAC
TN7 sec for (genome)	CCGAACAACGAATTGCTGGAAAA
TN7 sec rev (genome)	GTCAGGCTTGCTTCTGC
pBAD_sense	GGTGATGTCGGCGATATAGG
FLP_rev_	GAGCTCTTCTTATATGCGTCTATTTATGTAGGATGAAAGG
FRT_Cassett as	P- CCCGGGGATATCACTAGTGAAGT
FRT_Cassett_sense	AGGCCTCATATGGAATTCGGTACC
pBAD_as	CCACACACCATAGCTTCAAAATG
pSG1_his_Rev	P-GTGGTGGTGATGATGATGTGCCA
pSG1_rev_gg	ggctaccacctgctagtTTGTGGCATAGTGGTGGTGATGATGATGTGC
pSGFF_FRT_PaqCI_sense	AGGAACTTCCACCTGC
psGFF_FRT_PaqCI_as	GACACCTGCAGCAGCGAAGTTCCTATACTATTTGAAGAATAGG
pSIM6_ori_as	P-TATAAGAGACAGCTGACGGGTTTTG
pSIM6_ori_sense	P-CATAAAAGGCGCCTGTAGTGC
pSG1_fw_gg	ggctaccacctgcgctaaAGAGTCGACCTGCAGGCATG
pSG1_rev_gg	ggctaccacctgctagtttgtggcataGTGGTGGTGATGATGATGTGC
pCP20_for_gg	ggctaccacctgcgctatctTTATATGCGTCTATTTATGTAGG
pCP20_rev_gg	ggctaccacctgctagtACAATTTGGTATATTATGTAAAACAC
pSG2_FLP_rev	TGCCATGGTGAATTCCTCC
pSG4_BB_TN7_as	ggctaccacctgctggagcgagaAGTTCCTATACTATTTGAAGAATAG

pSG4_BB_TN7 sense	ggctaccacctgcatgtgcctCCGCCTCGAGATAACTTC
TN7_PaqCI_s	gacacctgcgtgttcgCAGTAGTAGGTTGAGGCCGTTG
oFTN2 GG rev	ggctaccacctgcttcaaggcGCCAATCCCATGGCGGATATAG
TN7 SCS integration for	AGATGACGGTTTGTCACATGGAGTTGGCAGGATGTTTGATTAAAAACATAGGacgtcctgacaaaaaac
TN7 SCS integration rev	CAGCCGCGTAACCTGGCAAAATCGGTTACGGTTGAGTAATAAATGGATGCtgacgctcagtggaacgaa
FRT_insert_sense	AGATGACGGTTTGTCACATGGAGTTGGCAGGATGTTTGATTAAAAACATAGGCCTCATATGGAATTCGGTACC
FRT_insert_as	CAGCCGCGTAACCTGGCAAAATCGGTTACGGTTGAGTAATAAATGGATGCCCGGGGATATCACTAGTGAAGT

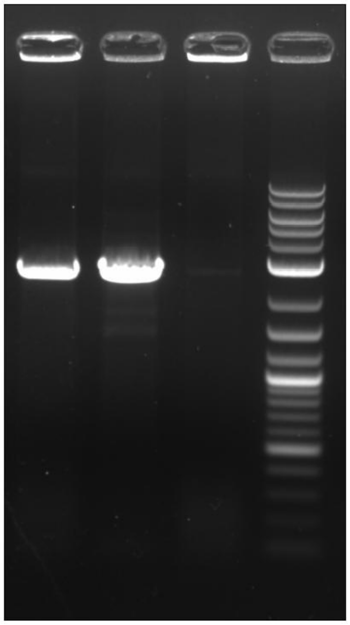

Supplementary Figure S1: Genome integrated sfGFP PCR verification after curing of pSG4 at day 10 of the protocol.

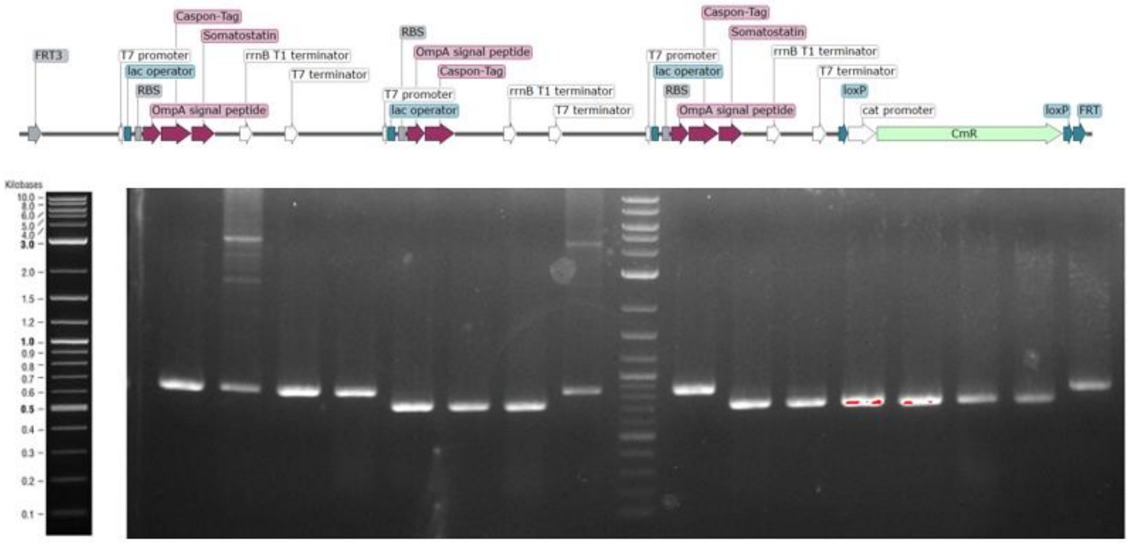

Supplementary Figure S2: Schematic illustration of the 3x_caspon_SST peptide fragment and screening of 16 colonies on day 4 of the RMCE genome integration protocol resulted in 2 out of 16 positive colonies of the 3x (caspon_SST) construct.

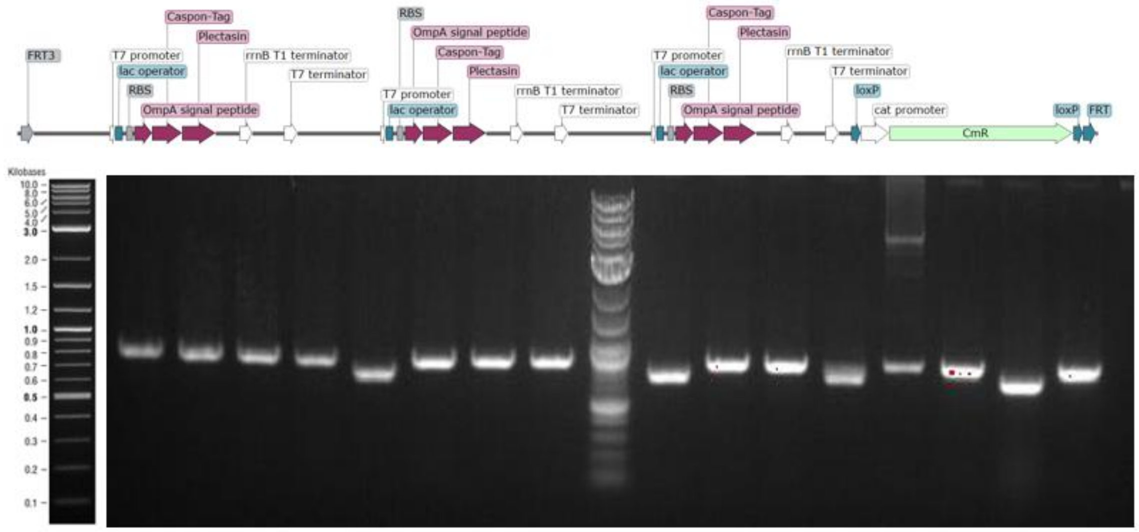

Supplementary Figure S3: Schematic illustration of the 3x_caspon_PLEC peptide fragment. Screening of 16 colonies on day 4 of the RMCE genome integration protocol resulted in 1 out of 16 positive colonies of the 3x (caspon_PLEC) construct.

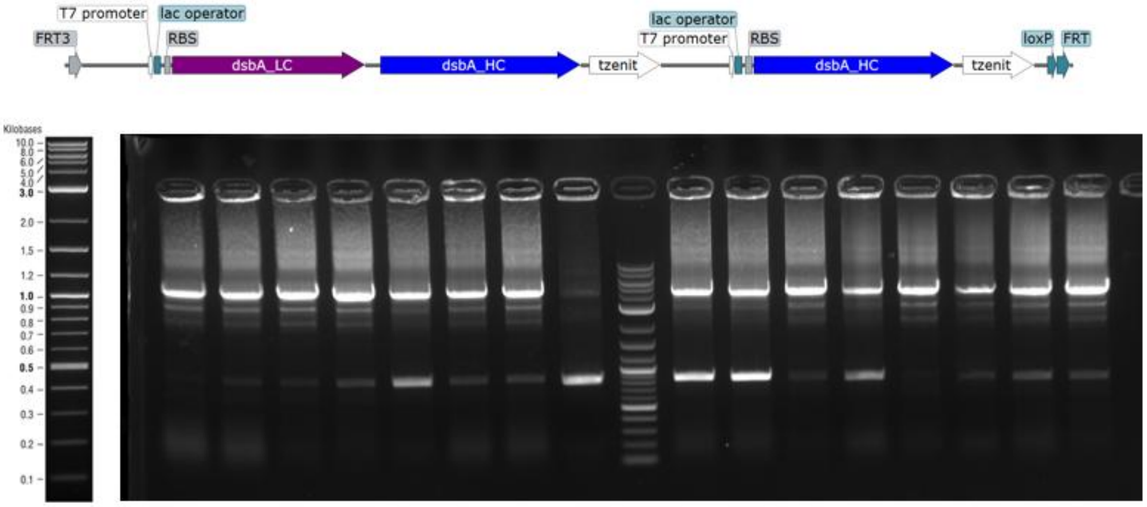

Supplementary Figure S4: Schematic illustration of the Fab2 T7_LC_HC + T7_HC fragment. Screening of 16 colonies on day 4 of the RMCE genome integration protocol resulted in 15 out of 16 positive colonies of the Fab2 T7_LC_HC + T7_HC construct.

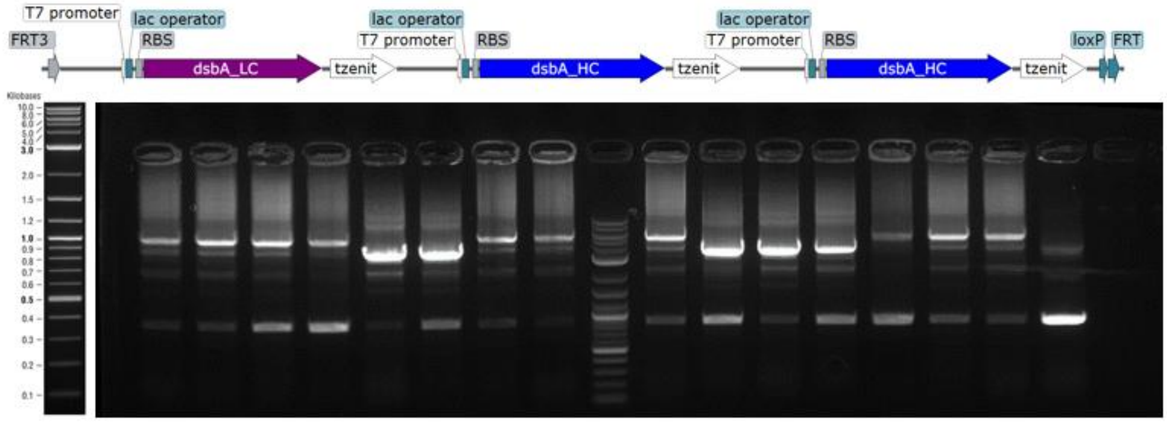

Supplementary Figure S5: Schematic illustration of the Fab3 T7_LC T7_HC + T7_HC fragment. Screening of 16 colonies on day 4 of the RMCE genome integration protocol resulted in 10 out of 16 positive colonies of the Fab3 T7_LC T7_HC + T7_HC construct.

Sequence of linear pSG4 with Esp3I GGA overhangs for cloning:

ggctaccgtctccagtactgcgatgagtggcAGGGCGGGGCGTGAATAACTTCGTATAGCATACATTATACGAAGTTATGAAGTTCCTATTCCG
AAGTTCCTATTCTCTAGAAAGTATAGGAACTTCACTAGTGATATCCCCGGGGCTTTCAGCCGGCAAACCGGCTGAAGCCGGATC
TGCGATTCTGATAACAAACTAGCAACACCAGAACAGCCCGTTTGCGGGCAGCAAAACCCGTCAGCTGTCTCTTATAGGTGATGT
CGGCGATATAGGCGCCAGCAACCGCACCTGTGGCGCCGGTGATGCCGGCCACGATGCGTCCGGCGTAGAGGATCTGCTCATG
TTTGACAGCTTATCATCGATGCATAATGTGCCTGTCAAATGGACGAAGCAGGGATTCTGCAAACCCTATGCTACTCCGTCAAGCC
GTCAATTGTCTGATTCGTTACCAATTATGACAACTTGACGGCTACATCATTCACTTTTTCTTCACAACCGGCACGGAACTCGCTCG
GGCTGGCCCCGGTGCATTTTTTAAATACCCGCGAGAAATAGAGTTGATCGTCAAAACCAACATTGCGACCGACGGTGGCGATAG
GCATCCGGGTGGTGCTCAAAAGCAGCTTCGCCTGGCTGATACGTTGGTCCTCGCGCCAGCTTAAGACGCTAATCCCTAACTGCT
GGCGGAAAAGATGTGACAGACGCGACGGCGACAAGCAAACATGCTGTGCGACGCTGGCGATATCAAAATTGCTGTCTGCCAGG
TGATCGCTGATGTACTGACAAGCCTCGCGTACCCGATTATCCATCGGTGGATGGAGCGACTCGTTAATCGCTTCCATGCGCCGC
AGTAACAATTGCTCAAGCAGATTTATCGCCAGCAGCTCCGAATAGCGCCCTTCCCCTTGCCCGGCGTTAATGATTTGCCCAAAC
AGGTCGCTGAAATGCGGCTGGTGCGCTTCATCCGGGCGAAAGAACCCCGTATTGGCAAATATTGACGGCCAGTTAAGCCATTC
ATGCCAGTAGGCGCGCGGACGAAAGTAAACCCACTGGTGATACCATTCGCGAGCCTCCGGATGACGACCGTAGTGATGAATCT
CTCCTGGCGGGAACAGCAAAATATCACCCGGTCGGCAAACAAATTCTCGTCCCTGATTTTTCACCACCCCCTGACCGCGAATGG
TGAGATTGAGAATATAACCTTTCATTCCCAGCGGTCGGTCGATAAAAAAATCGAGATAACCGTTGGCCTCAATCGGCGTTAAACC
CGCCACCAGATGGGCATTAAACGAGTATCCCGGCAGCAGGGGATCATTTTGCGCTTCAGCCATACTTTTCATACTCCCGCCATT
CAGAGAAGAAACCAATTGTCCATATTGCATCAGACATTGCCGTCACTGCGTCTTTTACTGGCTCTTCTCGCTAACCAAACCGGTA
ACCCCGCTTATTAAAAGCATTCTGTAACAAAGCGGGACCAAAGCCATGACAAAAACGCGTAACAAAAGTGTCTATAATCACGGCA
GAAAAGTCCACATTGATTATTTGCACGGCGTCACACTTTGCTATGCCATAGCATTTTTATCCATAAGATTAGCGGATCCTACCTGA
CGCTTTTTATCGCAACTCTCTACTGTTTCTCCATACCCGTTTTTTTGGGCTAGCAGGAGGATATACATATGCATCAAAAAAACCAG
GTAATGAACCTGGGTCCGAACTCTAAACTGCTGAAAGAATACAAATCCCAGCTGATCGAACTGAACATCGAACAGTTCGAAGCA
GGTATCGGTCTGATCCTGGGTGATGCTTACATCCGTTCTCGTGATGAAGGTAAAACCTACTGTATGCAGTTCGAGTGGAAAAACA
AAGCATACATGGACCACGTATGTCTGCTGTACGATCAGTGGGTACTGTCCCCGCCGCACAAAAAACAACGTGTTAACCACCTGG
GTAACCTGGTAATCACCTGGGGCGCCCAGACTTTCAAACACCAAGCTTTCAACAAACTGGCTAACCTGTTCATCGTTAACAACAA
AAAAACCATCCCGAACAACCTGGTTGAAAACTACCTGACCCCGATGTCTCTGGCATACTGGTTCATGGATGATGGTGGTAAATG
GGATTACAACAAAAACTCTACCAACAAATCGATCGTACTGAACACCCAGTCTTTCACTTTCGAAGAAGTAGAATACCTGGTTAAG
GGTCTGCGTAACAAATTCCAACTGAACTGTTACGTAAAAATCAACAAAAACAAACCGATCATCTACATCGATTCTATGTCTTACCT
GATCTTCTACAACCTGATCAAACCGTACCTGATCCCGCAGATGATGTACAAACTGCCGAACACTATCTCCTCCGAAACTTTCCTG
AAATAAGAATTCGTGTAGGAGGAATTCACCAGAGTCTCGAAAATAGGAGGTTTTTTTATGCCACAATTTGGTATATTATGTAAAAC
ACCACCTAAGGTGCTTGTTCGTCAGTTTGTGGAAAGGTTTGAAAGACCTTCAGGTGAGAAAATAGCATTATGTGCTGCTGAACTA
ACCTATTTATGTTGGATGATTACACATAACGGAACAGCAATCAAGAGAGCCACATTCATGAGCTATAATACTATCATAAGCAATTC
GCTGAGTTTCGATATTGTCAATAAATCACTCCAGTTTAAATACAAGACGCAAAAAGCAACAATTCTGGAAGCCTCATTAAAGAAAT
TGATTCCTGCTTGGGAATTTACAATTATTCCTTACTATGGACAAAAACATCAATCTGATATCACTGATATTGTAAGTAGTTTGCAAT
TACAGTTCGAATCATCGGAAGAAGCAGATAAGGGAAATAGCCACAGTAAAAAAATGCTTAAAGCACTTCTAAGTGAGGGTGAAAG
CATCTGGGAGATCACTGAGAAAATACTAAATTCGTTTGAGTATACTTCGAGATTTACAAAAACAAAAACTTTATACCAATTCCTCTT
CCTAGCTACTTTCATCAATTGTGGAAGATTCAGCGATATTAAGAACGTTGATCCGAAATCATTTAAATTAGTCCAAAATAAGTATCT
GGGAGTAATAATCCAGTGTTTAGTGACAGAGACAAAGACAAGCGTTAGTAGGCACATATACTTCTTTAGCGCAAGGGGTAGGAT
CGATCCACTTGTATATTTGGATGAATTTTTGAGGAATTCTGAACCAGTCCTAAAACGAGTAAATAGGACCGGCAATTCTTCAAGCA
ATAAACAGGAATACCAATTATTAAAAGATAACTTAGTCAGATCGTACAATAAAGCTTTGAAGAAAAATGCGCCTTATTCAATCTTTG
CTATAAAAAATGGCCCAAAATCTCACATTGGAAGACATTTGATGACCTCATTTCTTTCAATGAAGGGCCTAACGGAGTTGACTAAT
GTTGTGGGAAATTGGAGCGATAAGCGTGCTTCTGCCGTGGCCAGGACAACGTATACTCATCAGATAACAGCAATACCTGATCAC
TACTTCGCACTAGTTTCTCGGTACTATGCATATGATCCAATATCAAAGGAAATGATAGCATTGAAGGATGAGACTAATCCAATTGA
GGAGTGGCAGCATATAGAACAGCTAAAGGGTAGTGCTGAAGGAAGCATACGATACCCCGCATGGAATGGGATAATATCACAGG
AGGTACTAGACTACCTTTCATCCTACATAAATAGACGCATATAAAGAGTCGACCTGCAGGCATGCAAGCTTGGCTGTTTTGGCGG
ATGAGAGAAGATTTTCAGCCTGATACAGATTAAATCAGAACGCAGAAGCGGTCTGATAAAACAGAATTTGCCTGGCGGCAGTAG
CGCGGTGGTCCCACCTGACCCCATGCCGAACTCAGAAGTGAAACGCCGTAGCGCCGATGGTAGTGTGGGGTCTCCCCATGCG

AGAGTAGGGAACTGCCAGGCATCAAATAAAACGAAAGGCTCAGTCGAAAGACTGGGCCTTTCGTTTTATCTGTTGTTTGTCGGT
GAACGCTCTCCTGAGTAGGACAAATCCGCCGGGAGCGGATTTGAACGTTGCGAAGCAACGGCCCGGAGGGTGGCGGGCAGGA
CGCCCGCCATAAACTGCCAGGCATCAAATTAAGCAGAAGGCCATCCTGACGGATGGCCTTTTTGCGTTTCTACAAACTCTTTTGT
TTATTTTTCTAAATACATTCAAATATGTATCCGCTCATGAGACAATAACCCTGATAAATGCTTCAATAATATTGAAAAAGGAAGAGT
ATGAGTATTCAACATTTCCGTGTCGCCCTTATTCCCTTTTTTGCGGCATTTTGCCTTCCTGTTTTTGCTCACCCAGAAACGCTGGT
GAAAGTAAAAGATGCTGAAGATCAGTTGGGTGCACGAGTGGGTTACATCGAACTGGATCTCAACAGCGGTAAGATCCTTGAGAG
TTTTCGCCCCGAAGAACGTTTTCCAATGATGAGCACTTTTAAAGTTCTGCTATGTGGCGCGGTATTATCCCGTGTTGACGCCGGG
CAAGAGCAACTCGGTCGCCGCATACACTATTCTCAGAATGACTTGGTTGAGATCGACAAAGGAAAAGGGGGACGGATCTCCGA
GGCCTCGGACCCGTCGGGCCGCGTCGGACGTGTCAGTCCTGCTCCTCGGCCACGAAGTGCACGCAGTTGCCGGCCGGGTCG
CGCAGGGCGAACTCCCGCCCCCACGGCTGCTCGCCGATCTCGGTCATGGCCGGCCCGGAGGCGTCCCGGAAGTTCGTGGAC
ACGACCTCCGACCACTCGGCGTACAGCTCGTCCAGGCCGCGCACCCACACCCAGGCCAGGGTGTTGTCCGGCACCACCTGGT
CCTGGACCGCGCTGATGAACAGGGTCACGTCGTCCCGGACCACACCGGCGAAGTCGTCCTCCACGAAGTCCCGGGAGAACCC
GAGCCGGTCGGTCCAGAACTCGACCGCTCCGGCGACGTCGCGCGCGGTGAGCACCGGAACGGCACTGGTCAACTTGGCCAT
GGTTTAGTTCCTCACCTTGTCGTATTATACTATGCCAATATACTATGCCGATGATTAATTGTCAACACCGCCCTTAGATTAGATTG
CTATGCTTTCTTTCTAATGAGCAAGAAGTAAAAAAAGTTGTAATAGAACAAGAAAAATGAAACTGAAACTTGAGAAATTGAAGACC
GTTTATTAACTTAAATATCAATGGGAGGTCATCGAAAGAGAAAAAAATCAAAAAAAAAAATTTTCAAGAAAAAGAAACGTGATAAA
AATTTTTATTGCCTTTTTCGACGAAGAAAAAGAAACGAGGCGGTCTCTTTTTTCTTTTCCAAACCTTTAGTACGGGTAATTAACGA
CACCCTAGAGGAAGAAAGAGGGGAAATTTAGTATGCTGTGCTTGGGTGTTTTGAAGTGGTACGGCGATGCGCGGAGTCCGAGA
AAATCTGGAAGAGTAAAAAAGGAGTAGAAACATTTTGAAGCTATGGTGTGTGGCATAAAAGGCGCCTGTAGTGCCATTTACCCCC
ATTCACTGCCAGAGCCGTGAGCGCAGCGAACTGAATGTCACGAAAAAGACAGCGACTCAGGTGCCTGATGGTCGGAGACAAAA
GGAATATTCAGCGATTTGCCCGAGCTTGCGAGGGTGCTACTTAAGCCTTTAGGGTTTTAAGGTCTGTTTTGTAGAGGAGCAAACA
GCGTTTGCGACATCCTTTTGTAATACTGCGGAACTGACTAAAGTAGTGAGTTATACACAGGGCTGGGATCTATTCTTTTTATCTTT
TTTTATTCTTTCTTTATTCTATAAATTATAACCACTTGAATATAAACAAAAAAAACACACAAAGGTCTAGCGGAATTTACAGAGGGT
CTAGCAGAATTTACAAGTTTTCCAGCAAAGGTCTAGCAGAATTTACAGATACCCACAACTCAAAGGAAAAGGACTAGTAATTATCA
TTGACTAGCCCATCTCAATTGGTATAGTGATTAAAATCACCTAGACCAATTGAGATGTATGTCTGAATTAGTTGTTTTCAAAGCAA
ATGAACTAGCGATTAGTCGCTATGACTTAACGGAGCATGAAACCAAGCTAATTTTATGCTGTGTGGCACTACTCAACCCCACGAT
TGAAAACCCTACAAGGAAAGAACGGACGGTATCGTTCACTTATAACCAATACGTTCAGATGATGAACATCAGTAGGGAAAATGCT
TATGGTGTATTAGCTAAAGCAACCAGAGAGCTGATGACGAGAACTGTGGAAATCAGGAATCCTTTGGTTAAAGGCTTTGAGATTT
TCCAGTGGACAAACTATGCCAAGTTCTCAAGCGAAAAATTAGAATTAGTTTTTAGTGAAGAGATATTGCCTTATCTTTTCCAGTTA
AAAAAATTCATAAAATATAATCTGGAACATGTTAAGTCTTTTGAAAACAAATACTCTATGAGGATTTATGAGTGGTTATTAAAAGAA
CTAACACAAAAGAAAACTCACAAGGCAAATATAGAGATTAGCCTTGATGAATTTAAGTTCATGTTAATGCTTGAAAATAACTACCA
TGAGTTTAAAAGGCTTAACCAATGGGTTTTGAAACCAATAAGTAAAGATTTAAACACTTACAGCAATATGAAATTGGTGGTTGATA
AGCGAGGCCGCCCGACTGATACGTTGATTTTCCAAGTTGAACTAGATAGACAAATGGATCTCGTAACCGAACTTGAGAACAACC
AGATAAAAATGAATGGTGACAAAATACCAACAACCATTACATCAGATTCCTACCTACATAACGGACTAAGAAAAACACTACACGAT
GCTTTAACTGCAAAAATTCAGCTCACCAGTTTTGAGGCAAAATTTTTGAGTGACATGCAAAGTAAGTATGATCTCAATGGTTCGTT
CTCATGGCTCACGCAAAAACAACGAACCACACTAGAGAACATACTGGCTAAATACGGAAGGATCTGAGGTTCTTATGGCTCTTGT
ATCTATCAGTGAAGCATCAAGACTAACAAACAAAAGTAGAACAACTGTTCACCGTTACATATCAAAGGGAAAACTGTCCATATGCA
CAGATGAAAACGGTGTAAAAAAGATAGATACATCAGAGCTTTTACGAGTTTTTGGTGCATTCAAAGCTGTTCACCATGAACAGAT
CGACAATGTAACAGATGAACAGCATGTAACACCTAATAGAACAGGTGAAACCAGTAAAACAAAGCAACTAGAACATGAAATTGAA
CACCTGAGACAACTTGTTACAGCTCAACAGTCACACATAGACAGCCTGAAACAGGCGATGCTGCTTATCGAATCAAAGCTGCCG
ACAACACGGGAGCCAGTGACGCCTCCCGTGGGGAAAAAATCATGGCAATTCTGGAAGAAATAGCAGGCCTCATATGGAATTCG
GTACCACCGGTGAAGTTCCTATTCCGAAGTTCCTATTCTTCAAATAGTATAGgaacttcgcagtagtaggttgagagacggtagcc

FRT1 = green FRT2= red ORI = yellow FLP = blue

Sequence of FRT sites located on the genome of BL21(DE3)::FRT_RS I-SceI_FRT3:

GAAGTTCCTATTCCGAAGTTCCTATTCTTCAAATAGTATAGGAACTTCCACCTGCGCGTAGGGATAACAGGGTAATCGCCTCGAG
ATAACTTCGTATAGCATACATTATACGAAGTTATGAAGTTCCTATTCCGAAGTTCCTATTCTCTAGAAAGTATAGGAACTTC

Sequence of the integration fragment with sfGFP:

GAAGTTCCTATTCCGAAGTTCCTATTCTTCAAATAGTATAGGAACTTCgcAGTAGTAGGTTGAGGCCGTTGAGCACCGCCGCCGC
AAGGAATGGTGCATGCAAGGAGATGGCGCCCAACAGTCCCCCGGCCACGGGGCCTGCCACCATACCCACGCCGAAACAAGCG
CTCATGAGCCCGAAGTGGCGAGCCCGATCTTCCCCATCGGTGATGTCGGCGATATAGGCGCCAGCAACCGCACCTGTGGCGC
CGGTGATGCCGGCCACGATGCGTCCGGCGTAGAGGATCGAGATCGATCTCGATCCCGCGAAATTAATACGACTCACTATAGGG
GAATTGTGAGCGGATAACAATTCCCCTCTAGAAATAATTTTGTTTAACTTTAAGAAGGAGATATACATATGAAAAAGATTTGGCTG
GCGCTGGCTGGTTTAGTTTTAGCGTTTAGCGCATCGGCGCGTAAAGGCGAAGAGCTGTTCACTGGTGTCGTCCCTATTCTGGTG
GAACTGGATGGTGATGTCAACGGTCATAAGTTTTCCGTGCGTGGCGAGGGTGAAGGTGACGCAACTAATGGTAAACTGACGCT
GAAGTTCATCTGTACTACTGGTAAACTGCCGGTACCTTGGCCGACTCTGGTAACGACGCTGACTTATGGTGTTCAGTGCTTTGCT
CGTTATCCGGACCATATGAAGCAGCATGACTTCTTCAAGTCCGCCATGCCGGAAGGCTATGTGCAGGAACGCACGATTTCCTTT
AAGGATGACGGCACGTACAAAACGCGTGCGGAAGTGAAATTTGAAGGCGATACCCTGGTAAACCGCATTGAGCTGAAAGGCAT
TGACTTTAAAGAAGACGGCAATATCCTGGGCCATAAGCTGGAATACAATTTTAACAGCCACAATGTTTACATCACCGCCGATAAA
CAAAAAAATGGCATTAAAGCGAATTTTAAAATTCGCCACAACGTGGAGGATGGCAGCGTGCAGCTGGCTGATCACTACCAGCAA
AACACTCCAATCGGTGATGGTCCTGTTCTGCTGCCAGACAATCACTATCTGAGCACGCAAAGCGTTCTGTCTAAAGATCCGAAC
GAGAAACGCGATCATATGGTTCTGCTGGAGTTCGTAACCGCAGCGGGCATCACGCATGGTATGGATGAACTGTACAAATGATAA
CAATTAGGGGTCTCGCGGGGTTTTTTGCTGAAAGAAGCTTCAAATAAAACGAAAGGCTCAGTCGAAAGACTGGGCCTTTCGTTTT
ATCTGTTGTTTGTCGCTGCGGCCGCACTCGAGCACCACCACCACCACCACTGAGATCCGGCTGCTAACAAAGCCCGAAAGGAA
GCTGAGTTGGCTGCTGCCACCGCTGAGCAATAACTAGCATAACCCCTTGGGGCCTCTAAACGGGTCTTGAGGGGTTTTTTGCTG
AAAGGAGGAACTATATCCGGCCGCCTCGAGATAACGAAGTTCCTATTCCGAAGTTCCTATTCTCTAGAAAGTATAGGAACTTC

FRT1 = green FRT2= red T7 promotor = yellow sfGFP = green

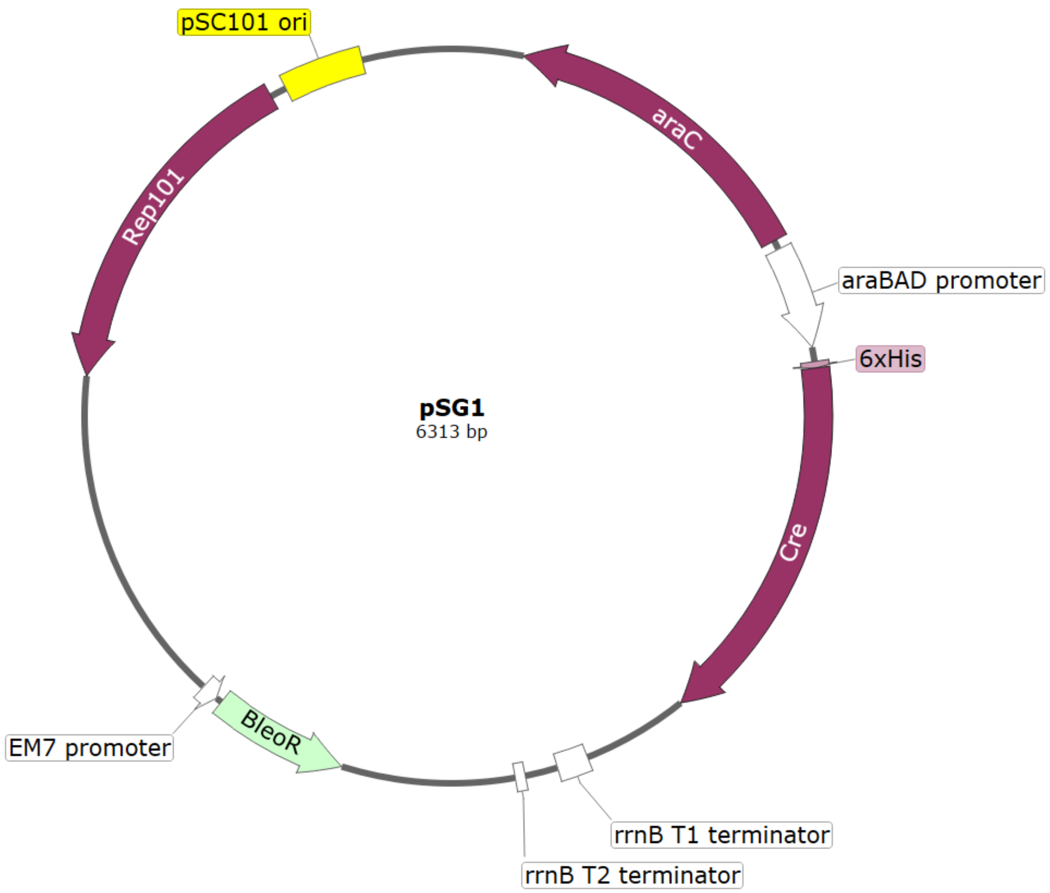

Supplementary Figure S6: Illustration of the pSG1 plasmid used for excision of loxP flanked CAT gene from the genome of BL21(DE3).
